# Cyclodextrin-Calcium Carbonate Micro- to Nano-Particles: Targeting Vaterite Form and Hydrophobic Drug Loading/Release

**DOI:** 10.3390/pharmaceutics15020653

**Published:** 2023-02-15

**Authors:** Cléa Chesneau, Alpha Oumar Sow, Fadila Hamachi, Laurent Michely, Séna Hamadi, Rémy Pires, André Pawlak, Sabrina Belbekhouche

**Affiliations:** 1 Université Paris Est Creteil, CNRS, Institut Chimie et Matériaux Paris Est, UMR 7182, 2 Rue Henri Dunant, F-94320 Thiais, France; 2 Université Paris Est, Faculté de Médecine, UMRS 955, Créteil, F-94010 France, UMRS 955, F-94010 Créteil, France; 3 Institut National de la Santé et de la Recherche Médicale (INSERM), IMRB U955, F-94010 Créteil, France

**Keywords:** cyclodextrin, hydrophobic drug, calcium carbonate particles, vaterite, drug delivery

## Abstract

Tailor-made and designed micro- and nanocarriers can bring significant benefits over their traditional macroscopic counterparts in drug delivery applications. For the successful loading and subsequent release of bioactive compounds, carriers should present a high loading capacity, trigger release mechanisms, biodegradability and biocompatibility. Hydrophobic drug molecules can accumulate in fat tissues, resulting in drawbacks for the patient’s recovery. To address these issues, we propose to combine the advantageous features of both host molecules (cyclodextrin) and calcium carbonate (CaCO_3_) particles in order to load hydrophobic chemicals. Herein, hybrid cyclodextrin-CaCO_3_ micro- to nano-particles have been fabricated by combining Na_2_CO_3_ solution and CaCl_2_ solution in the presence of an additive, namely poly (vinylsulfonic acid) (PVSA) or glycerol (gly). By investigating experimental parameters and keeping the Na_2_CO_3_ and CaCl_2_ concentrations constant (0.33 M), we have evidenced that the PVSA or gly concentration and mixing time have a direct impact on the final cyclodextrine-CaCO_3_ particle size. Indeed, by increasing the concentration of PVSA (5 mM to 30 mM) or gly (0.7 mM to 4 mM) or the reaction time (from 10 min to 4 h), particles with a size of 200 nm could be reached. Interestingly, the vaterite or calcite form could also be selected, according to the experimental conditions. We hypothesised that the incorporation of PVSA or gly into the precipitation reaction might reduce the nucleation rate by sequestering Ca^2+^. The obtained particles have been found to keep their crystal structure and surface charge after storage in aqueous media for at least 6 months. In the context of improving the therapeutic benefit of hydrophobic drugs, the developed particles were used to load the hydrophobic drug tocopherol acetate. The resulting particles are biocompatible and highly stable in a physiological environment (pH 7.4, 0.15 M NaCl). A selective release of the cargo is observed in acidic media (pH lower than 5).

## 1. Introduction

Nano- and even microparticles are extensively used as a delivery platform for biologically active materials [1]. Useful benefits of these particles include their stability when dispersed in aqueous media, their high drug loading capacity, the possibility to load either hydrophilic or hydrophobic chemicals or even both at once, and their sustained drug release. Efficient hydrophobic drugs are still of major interest for biological purposes, as their limited clinical effectiveness is mainly linked to low drug solubility in physiological media [2,3]. In comparison with conventional polymer or liposome containers, the unique characteristics of inorganic particles allow their increasing use in theranostic applications [4]. Liposomes are promising systems for drug delivery; however, they present the following disadvantages: low stability in the bloodstream, leading to leakage and fusion of the encapsulated drug, short half-life, possible oxidation and hydrolysis-like reactions of the phospholipid, and high production [5]. In the present work, special attention has been given to calcium carbonate (CaCO_3_) particles, as they have several benefits [6]. One may cite low cost, biocompatibility, pH sensitivity, easy fabrication without the use of organic solvents, and the ability to protect encapsulated material [7,8]. In this sense, the calcium carbonate system is an important material for both fundamental and applied study [9,10].

The fabrication of spherical monodispersed CaCO_3_ particles has been widely described in the literature [11,12]. Calcium carbonate can be present in three different anhydrous polymorphs: calcite, aragonite, and vaterite (written in order of decreasing thermodynamic stability) [13]. It is known that there is high interest in targeting the vaterite polymorph of calcium carbonate due to its higher porosity compared to the other polymorph forms and its peculiar optical and biochemical properties [14]. Vaterite CaCO_3_ particles have been proven to be efficiently used as biocompatible containers for the delivery of therapeutics into living cells and tissues [15]. Nevertheless, the thermodynamic instability of vaterite leads to its being naturally rare, and kinetic stabilisation is required to overcome this issue [16]. Several studies have thus investigated the effect of additive-directed crystallisation on CaCO_3_ crystal growth, targeting the vaterite form [17]. It was then noted that additional parameters, such as Ca^2+^ and CO_3_^2−^ concentrations, pH, temperature, mixing speed and reaction time greatly influence the size and polymorph form of the final CaCO_3_ particles. Interestingly, the addition of some chemical compounds, such as polymers [18,19], has been found to inhibit or stabilise the type of crystal structure depending on their concentration, charge density, or chemical composition [20]. For instance, poly (4-styrenesulfonate-co-maleic acid) was found to direct crystallisation to obtain a variety of superstructures [21], and a polyethyleneimine-assisted ultrasonic method was implemented for the fabrication of vaterite microparticles, which were stable for at least 8 months [22].

Much attention has been focused on the use of CaCO_3_ particles for biological purposes (e.g., drug delivery or sensor applications) [23]. Cell assays (i.e., the study of cell viability, efficient cellular uptake of chemical-loaded particles and cytotoxicity) have been used to investigate CaCO_3_ particles [24].

It is noted that such particles present the benefit of being pH sensitive [25], i.e., they are stable when suspended in a neutral environment, but are fully degraded in acidic media. CaCO_3_ particles have also been used as templates for the preparation of hollow particles (also called capsules) [8,26], e.g., polyelectrolyte capsules [8,27,28,29].

In the context of drug delivery, developing small vaterite calcium carbonate particles is of great interest as a drug-carrying particle; for instance, it is known that the particle size must be less than the 200–1200 nm pore size cut-off to passively go through a subcutaneous tumour cell [30]. It has been reported that the production of calcium carbonate particles presenting nanometric size requires the particle growth rate to be slowed down and the nanoparticles to be stabilised before their agglomeration leads to microparticles. In this sense, the well-known affinity of alginate chains towards calcium has been exploited to decrease the nucleation growth rate by sequestering calcium, resulting in the formation of calcium carbonate nanoparticles [31]. Nevertheless, the resulting CaCO_3_ nanoparticles present an increased size as well as a loss of negative surface charge after 4 h in aqueous media, disclosing surface recrystallisation. Ethylene glycol was employed in this sense and was found to lead to vaterite nanoparticles of up to 430 ± 10 nm in size [12]. These particles stayed in vaterite form in ethanol but recrystallised to the calcite form when suspended in aqueous solutions (e.g., in 0.9% w/v NaCl or in phosphate-buffered saline (PBS, 0.1 M, pH 7.4)). Smaller calcium carbonate particles have been obtained by adding poly (vinylsulfonic acid) (PVSA) (from 200 nm), which has been shown to promote the production of the vaterite form [13]. Indeed, it has also been previously reported that the presence of sulfonic groups on polymers stabilises the vaterite form [9]. The authors [13] evidenced that PVSA limits the interparticle bridging and aggregation of primary nuclei to limit/prevent microparticle formation. However, the resulting calcium carbonate particles presented a high polydispersity index. They used a PVSA with a high molecular weight of 4000–6000 kDa. Thus, even though few examples report how calcium carbonate nanoparticles presenting the vaterite form could be produced, long-term stability issues of vaterite nanoparticles in aqueous media and problems with non-uniform dispersity have unfortunately been identified.

Host-guest interactions are recognised as ingenious systems allowing the loading of hydrophobic drugs. Common host chemicals include crown ethers, pillararenes, cucurbiturils or even low-toxicity cyclic saccharides, such as cyclodextrins (CDs), which allow different kinds of hydrophobic guests to be entrapped inside their hydrophobic cavity. This results in the increased solubilisation of high levels of hydrophobic chemicals [32]. In our study, CD has been chosen as the host molecule as it presents several features, such as being biodegradable/biocompatible, favouring the solubilisation of hydrophobic substances, and transporting and even stabilising drugs in physiological media [33]. The features of CD have also been combined with other drug carriers such as liposomes [34] or nanoparticles [35], to name but a few.

In this sense, we propose to exploit the advantages of CD and calcium carbonate particles. We thus report on a simple way to fabricate CD-CaCO_3_ nanoparticles in which the vaterite form is promoted by doping the calcium solution with either PVSA (with low molecular weight and low concentration compared to published papers) or glycerol (studying low concentrations compared to published papers) before mixing with carbonate solution. The effects of reaction time and the PVSA and glycerol concentration on particle size, morphology, surface charge, and crystalline structure have been investigated. We also show that we can still take advantage of the CD for loading hydrophobic drugs. Note that mesoporous silica nanoparticles (MSN) are commonly synthesised by surfactant-templated sol–gel reactions. In our study, we propose an alternative compared to the reported work on MSN-CD, where calcium carbonate particles are prepared by simple precipitation at room temperature. We are targeting the possibility of obtaining a tailor-made particle size presenting stimuli responsiveness.

## 2. Experimental Section

### 2.1. Materials

Cyclodextrin (β-CD, γ-CD, HP-γ-CD and HP-β-CD), tocopherol acetate, tocopherol linoleate, miglyol, poly(vinylsulfonic acid) 30% wt in water (molecular weight of 2 kDa to 5 kDa according to manufacturer’s specifications, 3 kDa was used for calculation) were purchased from Sigma-Aldrich (Saint-Quentin-Fallavier, France).

Calcium chloride (≥99%, CaCl_2_) and sodium carbonate (≥99%, Na_2_CO_3_) were purchased from VWR Chemical, (Rosny-sous-Bois, France). Glycerol (≥99%) was purchased from Alfa Aesar (Illkirch-Graffenstaden, France). Water was purified with a Milli-Q reagent system (Millipore, Fontenay sous Bois, France).

### 2.2. Fabrication of Cyclodextrin-CaCO_3_ Particles

First of all, 10 mL of calcium chloride solution (CaCl_2_, 0.33 mol/L in water, 367 mg in 10 mL of water) containing CD (5 mg/mL) was mixed with 10 mL of sodium carbonate solution (Na_2_CO_3_, 0.33 mol/L, 350 mg in 10 mL of water) at 25 °C under vigorous stirring. This mixing leads to the rapid formation of CaCO_3_ microparticles due to the precipitation phenomenon. All the residual ions were then removed via a centrifugation step (9000 rpm, 4 min). The particles were then washed three times with water and finally dried at 80 °C over 24 h. The resulting sample was named “CD-CaCO_3_”.

### 2.3. Influence of the Addition of Either PVSA or Glycerol to Cyclodextrin-CaCO_3_ Particles

**Figure 1:**
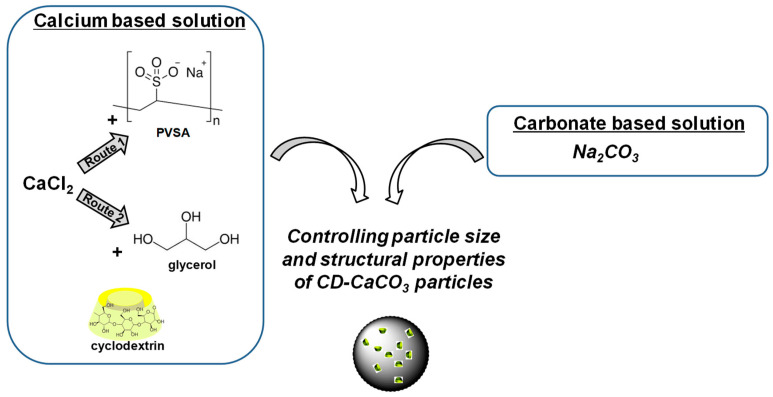
Scheme showing the process of fabrication of CD-calcium carbonate particles in presence of additive.

PVSA or glycerol was added to the calcium chloride solution, keeping the CaCl_2_ concentration constant (i.e., 0.33 mol/L). The impact of the amount of either PVSA or glycerol was investigated (Figure 1). In this sense, 10 mL of calcium chloride solution containing CD (5 mg/mL) was prepared in the presence of an additive (a mixture of CaCl_2_ solution at 0.33 mol/L to which either PVSA or glycerol was added) by stirring the CaCl_2_/CD/additive solution at 700 rpm for 30 min and mixing it with 10 mL of sodium carbonate solution (Na_2_CO_3_, 0.33 mol/L, 350 mg in 10 mL of water and allowing the reaction with Na_2_CO_3_ to occur under constant stirring at room temperature during either 10 min or 4 h). The following PVSA concentrations were studied: 30 mM, 20 mM, 10 mM and 5 mM. The following glycerol concentrations were studied: 4 mM, 3 mM, 1 mM and 0.7 mM. The samples were named as follows: “name of the additive_concentration of the additive_mixing time”. For instance, a sample resulting from the mixing of carbonate solution and calcium solution containing PVSA at a concentration of 30 mM and then mixed for 10 min would be named “CD-PVSA_30 mM_10 min”. Note that the same protocol for washing was used as in Section 2.2.

### 2.4. Characterisation of Hybrid Cyclodextrin-CaCO_3_ Particles

Scanning electron microscopy (SEM) was performed on a LEO1530 microscope equipped with an InLens and secondary electron detectors using a low accelerating voltage (3 kV) and was used to observe the shape of the obtained materials (in Leo Elektronenmikroskopie GmbH, Oberkochen, Germany).

Zeta potential measurements were performed on a Zetasizer Nano-ZS (Zetasizer 4700 Malvern Instruments, Palaiseau, France) with a suspension of CaCO_3_ particles (suspended in water).

Structural characterisation was performed by X-ray diffraction (XRD) using a D8 advance diffractometer (Bruker, Champs sur Marne, France) (Cu Kα radiation). Data were recorded over a 2Ɵ range from 5 to 80° in steps of 0.02° at an incident wavelength 𝜆 of 1.54056 Å.

Thermogravimetric analysis (TGA) was performed on a Setaram Setsys Evolution 16 thermobalance (Setaram Instrumentation, Caluire, France) by heating the samples at a rate of 10 °C/min from 30 to 900 °C.

The statistical analyses were performed with GraphPad Prism software, version 5.01 (USA) statistical package. Data were analysed using the Kruskal–Wallis test (*p* was always lower than 0.01).

### 2.5. Hydrophobic Loading of Hybrid CaCO_3_ Particles and Release

First of all, 10 mg of hybrid CaCO_3_ particles was mixed in a solution of tocopherol acetate and stirred at 700 rpm for 24 h at room temperature. The final concentration of the particle was 10 g/L. The resulting particles were washed with water, and the unloaded chemical was removed by centrifugation (7000 rpm). To determine the amount of tocopherol acetate loaded, the hybrid CaCO_3_ particles were destroyed at pH 2, and the concentration of tocopherol acetate was determined by absorbance measurements at 292 nm.

To monitor the tocopherol acetate release behaviour at room temperature, the chemical-loaded calcium carbonate particles were placed in a dialysis bag (3.5 kDa) and immersed in solution at pH 5.0 (200 mL). At predetermined intervals, 500 µL of the solution outside the dialysis bag was taken out and replaced with 500 µL of fresh buffer solution. The concentration of tocopherol acetate was determined by absorbance measurements at 292 nm.

### 2.6. Toxicity Test

Human embryonic kidney (HEK293; purchased from ATCC, ref CRL-1573) cells cultured at 60–80% confluence were used for the toxicity test. After exposure to hybrid CD-calcium carbonate particles for 48 h, human embryonic kidney (HEK293) cells were harvested, and proteins were extracted and analysed by Western blot with a specific antibody, cleaved caspase 3 (#9661, Cell Signaling Technology, Danvers, MA, USA). As a positive control for apoptosis, 20% dimethyl sulfoxide (DMSO) was added to the medium.

## 3. Results and Discussion

As presented in Figure 1, we aimed to synthesise hybrid cyclodextrin CD-CaCO_3_ particles via a co-precipitation technique. This approach is a simple and efficient method consisting of the direct mixing of soluble salts containing carbonate (CO_3_^2−^) and calcium (Ca^2+^). The impact of PVSA or glycerol additives on the resulting particle size and polymorph form was also investigated. The concentrations of additives in the calcium solution and the mixing time during the precipitation reaction were varied in order to understand how the particle size and morphology are affected.

**Figure 2:**
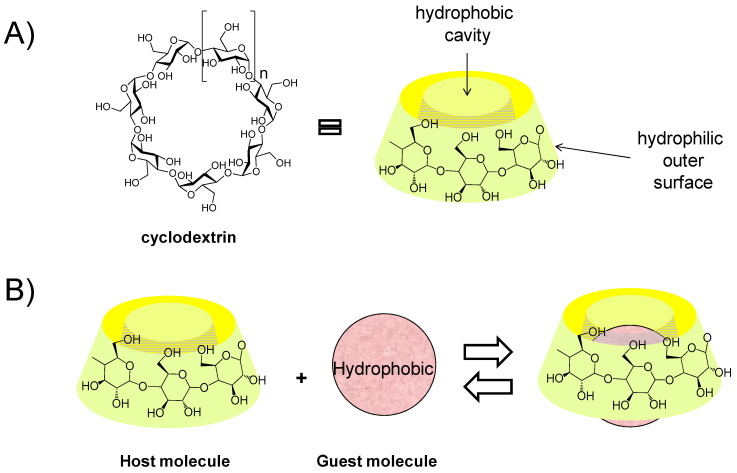
(**A**) chemical structure of β-cyclodextrin and (**B**) exchange in host–guest systems implying cyclodextrin.

It was noted that CD had no impact on the CaCO_3_ particles under the studied conditions. CD was used to fabricate particles, favouring the entrapment of hydrophobic molecules inside the particles. We employed β-cyclodextrin from the class of cyclic oligosaccharides with seven D-glucose units linked by β-1,4-glucose bonds (Figure 2A). This CD is water-soluble, nontoxic and commercially available. Most importantly, it has a hydrophobic cavity that can allow the loading of different hydrophobic molecules in an aqueous environment via the formation of host-guest inclusion complexes (Figure 2A) [36,37,38]. For more insight into the CD derivative implied in the assembly, the reader may refer to the review of Chen and Jiang [39]. In the biomedical field, the CD has proven stability and increases the aqueous solubility, bioavailability, and local tolerance of hydrophobic chemicals [40,41]. Finally, we investigated the possibility of entrapping hydrophobic tocopherol acetate inside the particles. The release of the loaded chemical cargo was studied in an acid environment.

The mixing of equal volumes of equimolar solutions of Na_2_CO_3_ and CaCl_2_ (10 mL each, 0.33 M) in the presence of CD without any additive (PVSA or glycerol) leads to an immediate increase in turbidity in the solution. This result shows the rapid formation of CD-CaCO_3_ particles. The SEM images of these particles are shown in Figure S1. As seen, these particles present a uniform and spherical shape with a rather narrow size distribution, from 4 to 6 µm. The zeta potential value of the CaCO_3_ particles suspended in water is −5 mV, i.e., they present negative surface charges.

**Figure 3:**
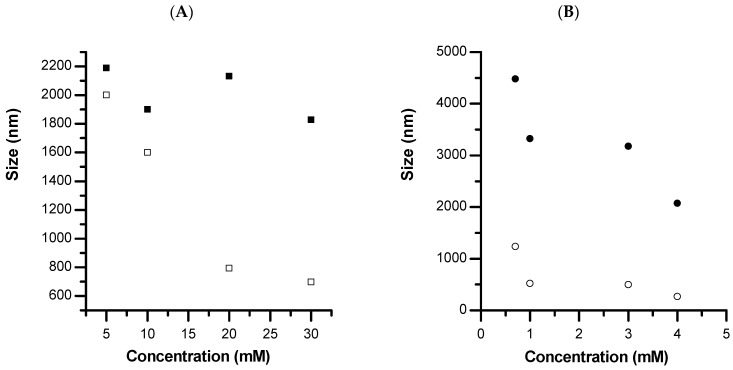
Size of CD-CaCO_3_ particles obtained in presence of (**A**) ■ PVSA (time of precipitation reaction: 10 min), □ PVSA (time of precipitation reaction: 4 h), (**B**) ● glycerol (time of precipitation reaction: 10 min), ○ glycerol (time of precipitation reaction: 4 h).

The inclusion of an additive, either PVSA or glycerol, was then investigated using different additive concentrations and stirring times. The concentration of PVSA (5 mM to 30 mM) or glycerol (0.7 mM to 4 mM) in the calcium solution was varied in order to study how the morphology and size of the calcium carbonate particle could be affected (Figure 3). A known limitation of DLS is the intensity-based measurement, which is weighted towards the higher scattering cross-section of particles presenting a large size. In this sense, the SEM technique was used to measure the size of larger particles and to assure the measured size of smaller particles. In the presence of PVSA, the particle size varies from 2200 nm to 1830 nm when the solutions are mixed for 10 min at room temperature. It is interesting to note that increasing the stirring time from 10 min to 4 h at room temperature decreases the particle size from 2000 nm to 700 nm when the concentration of PVSA is varied from 5 mM to 30 mM. The addition of PVSA to the particle process allows the nucleation rate to be lowered and the surface stabilised to avoid further growth or aggregation into microparticles.

The same observation can be made with the addition of glycerol, i.e., the addition of this compound induces a decrease in the size of the calcium carbonate particles, which vary from 4480 nm to 2070 nm when the concentration of glycerol varies from 0.7 mM to 4 mM and solutions are mixed for 10 min at room temperature. It is interesting to note that increasing the stirring time from 10 min to 4 h at room temperature decreases the particle size from 1230 nm to 260 nm when the glycerol concentration varies from 0.7 mM to 4 mM. Compared to the results published by authors Trushina et al. [15] who used glycerol to reduce the size of calcium carbonate particles, we find that we obtain comparable sizes by introducing much less glycerol. Indeed, they obtain sizes in the order of 300 nm with glycerol concentrations higher than 11 M, whereas we obtain the same order of magnitude with concentrations in the order of 4 mM. The big difference between their work and ours is the mixing time. They obtain nanometre-sized materials by adding much higher concentrations and stirring for 50 to 60 min. We also obtain similar sizes (in the order of 2 µm with 4 mM glycerol) by shaking the calcium and carbonate with additive solutions for only 10 min. However, by increasing the stirring time (from 10 min to 4 h) and working with low glycerol concentrations (from 1 mM), we obtain nanometric sizes. Working at low additive concentrations and using long mixing times seems to favour the formation of small particle sizes of calcium carbonate. Knowing that we are aiming for applications in the biomedical field, in order to overcome any cytotoxicity problems, it seems important to work at low additive concentrations. Based on the work by Trushina et al., the effect of glycerol is linked to a higher number of hydroxyl groups, providing more sites of nucleation, a stronger surface binding, and then stabilising the primary clusters of calcium carbonate [15].

Subsequently, these particles, whether prepared in the presence or absence of PVSA or glycerol, were suspended in water at 1 mg/mL and zeta potential measurements were performed. The value of this parameter is of the order of −5 mV for particles without the so-called “additive”, whereas the zeta potential value is of the order of −15 ± 2 mV in the presence of PVSA and of the order of −12 ± 2 mV in the presence of glycerol. The particles still present a negative surface charge for all PVSA or glycerol concentrations and remain negatively charged even after 6 months of storage, indicating that the PVSA or glycerol has stabilised the surface against aggregation. This result is in accordance with the results obtained by Nagaraja et al. [13]. There are two effects that can explain this variation compared to native CaCO_3_ particles. PVSA with -SO_3_^−^ groups (i.e., negatively charged PVSA embedded into the structure of particles) can be found on the surface and contribute to the measured negative zeta potential, in addition to the carbonate functions. The zeta potential also reflects colloidal stability. For small particles, having a decreasing zeta potential value may be consistent with an increase in the colloidal stability of the suspended particles. This last case is, from our point of view, the only explanation for the zeta potential values measured in the presence of glycerol.

**Figure 4:**
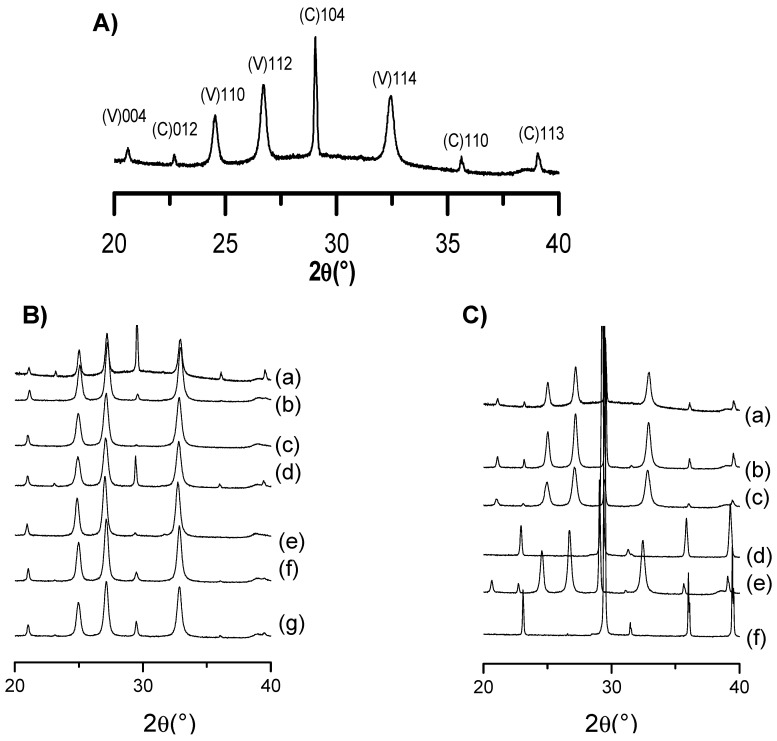
XRD patterns of (**A**) CD-CaCO_3_ particle, (**B**) CD-CaCO_3_ particle with PVSA and (**C**) CD-CaCO_3_ particle with glycerol. (**B**) (a) CD-CaCO3 microparticle, (b) CD-PVSA_30 mM_10min, (c) CD-PVSA_20 mM_10min, (d) CD-PVSA_10 mM_10min, (e) CD-PVSA_30 mM_4h, (f) CD-PVSA_20 mM_4h, (g) CD-PVSA_5 mM_4h. (**C**) (a) CD-CaCO3 microparticle, (b) CD-glycerol_3 mM_10min, (c) CD-glycerol_0.7 mM_10min, (d) CD-glycerol_4 mM_4h, (e) CD-glycerol_1 mM_4h, (f) CD-glycerol_4 mM_24h.

XRD analysis was additionally conducted to evaluate the different phase composition of CD-CaCO_3_ particles in the presence of the studied doping agent, i.e., in the presence of either PVSA or glycerol. Figure 4A presents the XRD pattern of the CaCO_3_ particles without the addition of PVSA or glycerol. As shown, vaterite and calcite are the main crystalline polymorphs, corresponding, respectively, to *hkl*: 004, 110, 112, 114 and 012, 104, 006, 110, 113. The XRD of CD-CaCO_3_ particles with either PVSA or glycerol additive are shown in Figure 4B,C and were analysed to investigate the possible impact on CaCO_3_ crystalline polymorphs. We evidence that calcite and vaterite are the main polymorphs present for all the designed CaCO_3_ particles; diffraction peaks are located at *hkl* indices (104) for calcite (C) and (110), (112) and (114) for vaterite (V).

**Figure 5:**
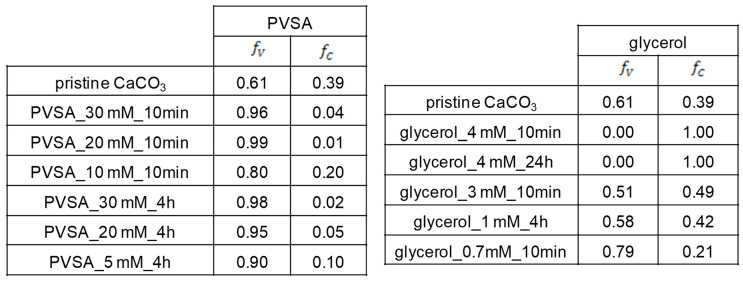
Relative fraction of vaterite and calcite in the hybrid CD-CaCO_3_ particle. f_v_: fraction of vaterite; f_c_: fraction of calcite.

From Rao’s equation (Equation (1)) [42], we can define the relative fractions of vaterite (f_v_) and calcite (f_c_) in each particle (Figure 5).

$$f_{V}=\frac{\left(I_{110V\;}+I_{112V\;}+I_{114V}\right)}{\left(I_{110V\;}+I_{112V\;}+I_{114V}+I_{104C}\right)}$$

**Figure 6:**
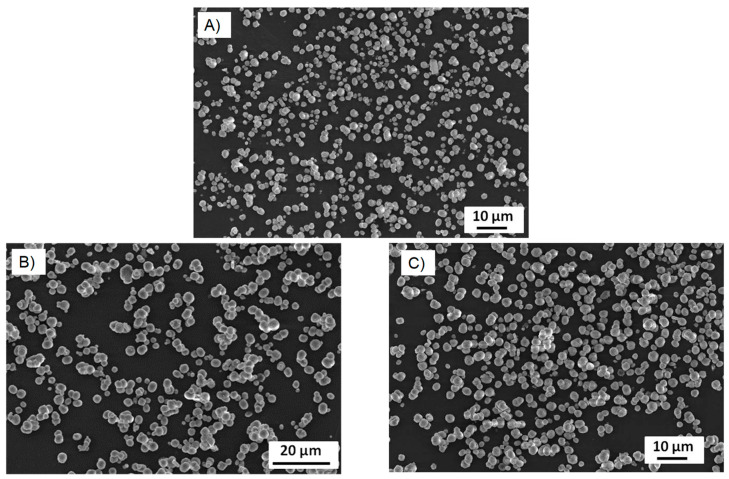
Scanning electron microscopy image (**A**) CD-PVSA_30mM_10min, (**B**) CD-PVSA_5mM_4h and (**C**) CD-PVSA_30mM_4h.

**Figure 7:**
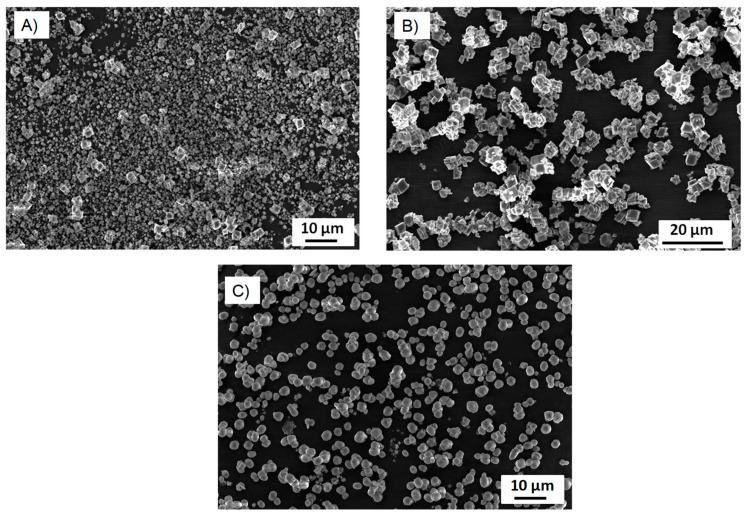
Scanning electron microscopy image (**A**) CD-glycerol_4mM_10min, (**B**) CD-glycerol_4mM_4h and (**C**) CD-glycerol_1mM_4h.

From the XRD results, we can see that the CD-PVSA-CaCO_3_ particles are predominantly in vaterite form (in most cases, over 90%) and that the rest are calcite. According to the data, the addition of PVSA into the coprecipitation medium leads to an increase in the vaterite phase compared to the reaction performed at the same molar ratio but without PVSA. These results are supported by the SEM images (Figure 6 and Figure 7). Indeed, calcium carbonate particles are observed to have a predominantly spherical morphology, which is consistent with a vaterite-type form, as reported by Nagaraja et al., who prepared calcium carbonate in the presence of PVSA but with a higher molecular weight (4000–6000 kDa) than in the present work (2 to 5 kDa) [13].

From the XRD results, we see that, for all incorporated glycerol concentrations, the calcite or vaterite form of CD-glycerol-CaCO_3_ particles is obtained according to the CaCO_3_ particle manufacturing process, and it is interesting to note that the desired polymorph form can be favoured, i.e., the vaterite form, depending on these conditions. For instance, the vaterite form is mainly obtained at glycerol concentrations of 0.7 mM and a mixing time of 10 min, and these particles have a nanometric size (measured in DLS and SEM). These results are corroborated by the SEM images (see Figure 7). To our knowledge, this result has not been previously reported. Indeed, when glycerol is added during the preparation of calcium carbonate particles, most studies report that the particles obtained are micrometric and mostly of the calcite form; when the vaterite form is obtained, it is not stable under the conditions obtained and changes to the calcite form (the unwanted form). Low concentrations of glycerol promote vaterite formation (0.7 mM to 1 mM), while increasing the glycerol concentration up to 3 M promotes calcite formation. The contributing characteristics of glycerol seem to be its ability to hydrogen bond and induce increased viscosity in the solution. At low glycerol concentrations, growth of the calcite nuclei is probably prevented by the glycerol forming an ion diffusion barrier due to its cited characteristics. It seems that increasing the concentration of glycerol inhibits this effect, favouring the calcite form.

**Figure 8:**
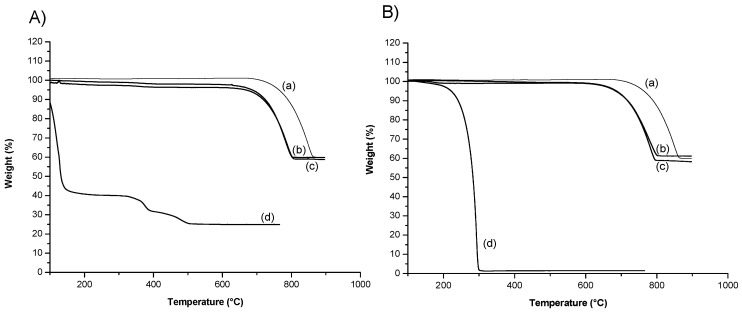
TGA curves of: (**A**) (a) CD-CaCO_3_, (b) CD-PVSA_30mM_10min, (c) CD-PVSA_30mM_4h, (d) PVSA and (**B**) (a) CD-CaCO_3_, (b) CD-glycerol_4mM_10min, (c) CD-glycerol_4mM_4h, (d) glycerol.

Next, we studied the thermal decomposition process of fabricated CD-CaCO_3_ particles by TGA. Figure 8 presents TGA curves for PVSA, the CD-CaCO_3_ microparticles, CD-CaCO_3_ particles with PVSA, glycerol, and CD-CaCO_3_ particles with glycerol. The CD-CaCO_3_ microparticles present a first loss of weight at 710 °C and lose about 40% of their weight above 800 °C. Free PVSA starts to lose weight at the beginning of the heating process. The CD-PVSA-CaCO_3_ particles start to lose weight above 700 °C, 690 °C higher than that of free PVSA. Free glycerol starts to lose weight at 180 °C and has lost its weight completely at 300 °C. The CD-glycerol-CaCO_3_ particles start to lose weight above 650 °C, 470 °C higher than that of free glycerol. These results are in agreement with those of Wang et al. [43], who loaded ibuprofen inside CaCO_3_ particles, and Belbekhouche et al., who loaded rhodamine b [29]. Based on their work, we can postulate that most of the PVSA or glycerol is mainly trapped inside the CD-CaCO_3_ microparticles and that the amount on the surface may be low/insignificant, which contributes to the negative charge of the particles, especially in the case of PVSA with SO_3_^−^ groups in addition to the carbonate part.

The toxicity of the particles was then evaluated according to a previously published protocol [44]. No difference was seen between the control, non-treated cells and the CD-calcium carbonate particle-treated cells, in either cell density or cell count. This result indicates a very low or non-existent toxic effect of the calcium carbonate particles. The toxicity data obtained are in agreement with previously published data: Bahrom et al. demonstrated an absence of cytotoxicity with differently shaped CaCO_3_ particles (spheroids, ellipsoids and toroids) on a C6 glioma cell model [45], and Zhang et al. evidenced the biocompatibility of porous CaCO_3_ particles in micrometre and nanometre ranges on HeLa cells [46].

Looking for a more effective hydrophobic drug delivery system, we propose to use the developed CD-CaCO_3_ particles to entrap and subsequently release hydrophobic chemicals. The pH sensitivity of the CaCO_3_ particles has been exploited to control tocopherol acetate according to the pH. Indeed, CaCO_3_ particles can be dissolved in an acidic environment (from pH 5) [47] and are stable in neutral media [12].

**Figure 9:**
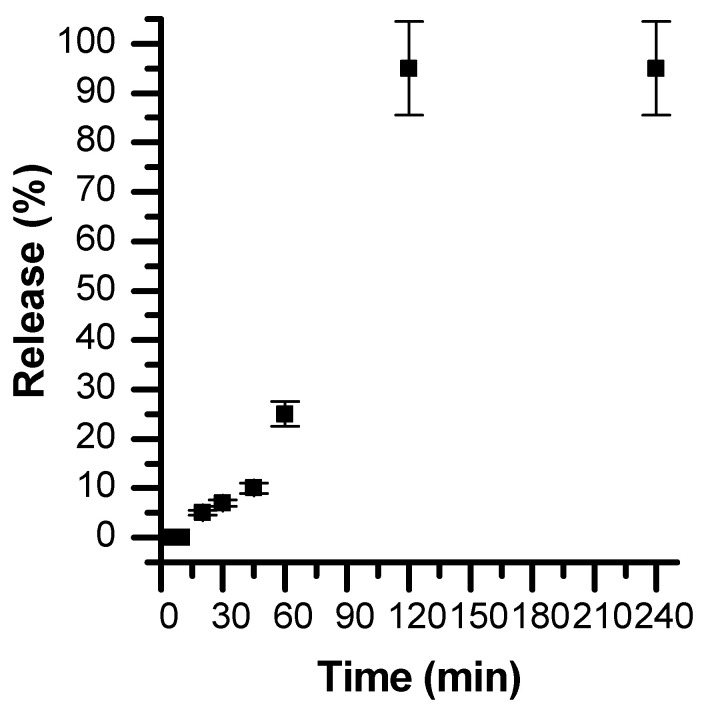
Tocopherol acetate release from CD-CaCO_3_ particles at pH 5.

**Figure 10:**
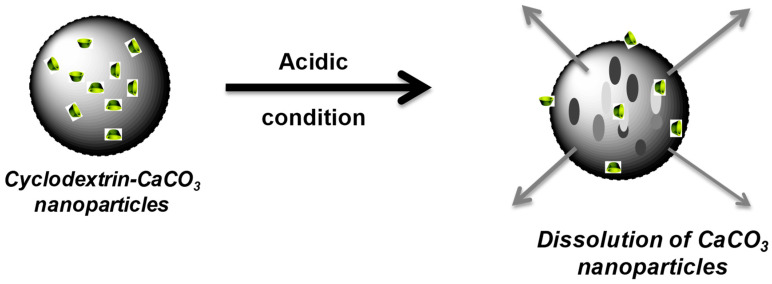
Schematic illustration of the dissolution of the hybrid CD-calcium carbonate particles in acidic media.

Tocopherol acetate was thus loaded inside the CD cavity of the CD-CaCO_3_ particles. To check the above assumption about the possible entrapment of β-CDs in the matrix of CaCO_3_ microparticles, a small amount of tocopherol acetate solubilised in ethanol was added to CaCO_3_ microparticles without the addition of β-CDs. It was found that 5.5 mg of tocopherol acetate was entrapped in a solution of 10 mg/mL CD-CaCO_3_ particles. However, less than 0.6 mg of tocopherol acetate was entrapped in a solution of 10 mg/mL CaCO_3_ particles without the addition of CD (CD-free particles). This result clearly highlights the efficiency of loading a hydrophobic drug using the hybrid CD-CaCO_3_ particles, which could be correlated to the formation of an inclusion complex between the tocopherol acetate molecule and b-CD in the matrix of CD-CaCO_3_ particles. The designed calcium carbonate particles were found to be highly stable at pH 7.4. However, when subjected to a slightly acidic medium, the particles are completely dissolved (within 1 h), leading to the consequent release of the hydrophobic substance (Figure 9 and Figure 10). This pH-dependent release of the hydrophobic compound from CaCO_3_ particles renders them suitable for the pH-triggered release of hydrophobic drugs in localised acidic areas (e.g., inside intracellular tumours and endosomes [48]).

The present study has been extended to other CD types (α-CD, γ-CD, HP-γ-CD and HP-β-CD), and we have also shown the possibility of loading either tocopherol linoleate or miglyol as hydrophobic molecules, instead of tocopherol acetate. We found the same efficiencies as tocopherol acetate in terms of trapping in CDs and release.

## 4. Conclusions

We addressed an easy and rapid approach to fabricating CD-calcium carbonate nanoparticles that was biocompatible and favoured the vaterite form. SEM images evidenced the particle formation, spherical morphology and vaterite form. This latter form has also been highlighted by XRD measurements. The proposed method does not require any specialised equipment, setup or organic solvent. The produced particle size and vaterite form can be selected by controlling either the PVSA or glycerol concentration and the mixing time during the precipitation phenomenon. The vaterite polymorph obtained according to the experimental conditions remains stable even after storing the particles in aqueous solution for up to 6 months. This is a crucial point, as most published methods fabricated vaterite calcium carbonate nanoparticles that were not stable in water [12,31]. Moreover, the designed CD-CaCO_3_ particles did not show any cytotoxicity. We also showed the possibility of using the resulting calcium carbonate particles for the loading of ionic hydrosoluble chemicals. Interestingly, at pH 7.4, the designed particles remain stable and no hydrophobic cargo release is observed; nevertheless, at pH 5, all the cargo is released because of calcium carbonate particle decomposition. Future experiments will be undertaken to further study cell uptake, intracellular distribution and intracellular drug release assays.

## Data Availability

Not applicable.

## Supplementary Materials

The following are available online at https://www.mdpi.com/article/10.3390/pharmaceutics15020653/s1, Figure S1: Scanning Electron Microscopy image of CD-calcium carbonate microparticles prepared.

## References

[1] Daum N., Tscheka C., Neumeyer A., Schneider M. (2012). Novel approaches for drug delivery systems in nanomedicine: Effects of particle design and shape. Wiley Interdiscip. Rev. Nanomed. Nanobiotechnol..

[2] Agarwal A., Lvov Y., Sawant R., Torchilin V. (2008). Stable nanocolloids of poorly soluble drugs with high drug content prepared using the combination of sonication and layer-by-layer technology. J. Control. Release.

[3] Smith R.C., Riollano M., Leung A., Hammond P.T. (2009). Layer-by-Layer Platform Technology for Small-Molecule Delivery. Angew. Chem. Int. Ed..

[4] Kang M.S., Kwon M., Jang H.J., Jeong S.J., Han D.-W., Kim K.S. (2022). Biosafety of inorganic nanomaterials for theranostic applications. Emergent Mater..

[5] Yadav D., Sandeep K., Pandey D., Dutta R.K. (2017). Liposomes for drug delivery. J. Biotechnol. Biomater..

[6] Belbekhouche S., Charaabi S., Carbonnier B. (2019). Glucose-sensitive capsules based on hydrogen-bonded (polyvinylpyrrolidone/phenylboronic –modified alginate) system. Colloids Surf. B Biointerfaces.

[7] Yang J., Zhang R., Pan H., Li Y., Fang Y., Zhang L., Kopeček J. (2017). Backbone Degradable *N*-(2-Hydroxypropyl)methacrylamide Copolymer Conjugates with Gemcitabine and Paclitaxel: Impact of Molecular Weight on Activity toward Human Ovarian Carcinoma Xenografts. Mol. Pharm..

[8] Belbekhouche S., Charaabi S., Picton L., Le Cerf D., Carbonnier B. (2018). Glucose-sensitive polyelectrolyte microcapsules based on (alginate/chitosan) pair. Carbohydr. Polym..

[9] Verdoes D., Van Landschoot R., Van Rosmalen G. (1990). Crystallization in detergent performance. J. Cryst. Growth.

[10] Cosentino I., Liendo F., Arduino M., Restuccia L., Bensaid S., Deorsola F., Ferro G.A. (2020). Nano CaCO_3_ particles in cement mortars towards developing a circular economy in the cement industry. Procedia Struct. Integr..

[11] Schmidt S., Volodkin D. (2013). Microparticulate biomolecules by mild CaCO_3_ templating. J. Mater. Chem. B.

[12] Parakhonskiy B.V., Haase A., Antolini R. (2012). Sub-Micrometer Vaterite Containers: Synthesis, Substance Loading, and Release. Angew. Chem. Int. Ed..

[13] Nagaraja A.T., Pradhan S., McShane M.J. (2014). Poly (vinylsulfonic acid) assisted synthesis of aqueous solution stable vaterite calcium carbonate nanoparticles. J. Colloid Interface Sci..

[14] Christy A.G. (2017). A Review of the Structures of Vaterite: The Impossible, the Possible, and the Likely. Cryst. Growth Des..

[15] Trushina D.B., Bukreeva T.V., Kovalchuk M.V., Antipina M.N. (2014). CaCO_3_ vaterite microparticles for biomedical and personal care applications. Mater. Sci. Eng. C.

[16] Wolf G., Königsberger E., Schmidt H.G., Königsberger L.-C., Gamsjäger H. (2000). Thermodynamic Aspects of the Vaterite-Calcite Phase Transition. J. Therm. Anal. Calorim..

[17] Naka K., Chujo Y. (2001). Control of Crystal Nucleation and Growth of Calcium Carbonate by Synthetic Substrates. Chem. Mater..

[18] Arias J.L., Neira-Carrillo A., Escobar C., Bodero M., David M., Fernández M.S. (2004). Sulfated polymers in biological mineralization: A plausible source for bio-inspired engineering. J. Mater. Chem..

[19] Butler M.F., Glaser N., Weaver A.C., Kirkland M., Heppenstall-Butler M. (2006). Calcium Carbonate Crystallization in the Presence of Biopolymers. Cryst. Growth Des..

[20] Song R.-Q., Cölfen H., Xu A.-W., Hartmann J., Antonietti M. (2009). Polyelectrolyte-Directed Nanoparticle Aggregation: Systematic Morphogenesis of Calcium Carbonate by Nonclassical Crystallization. ACS Nano.

[21] Song R.-Q., Xu A.-W., Antonietti M., Cölfen H. (2009). Calcite Crystals with Platonic Shapes and Minimal Surfaces. Angew. Chem. Int. Ed..

[22] López-Marzo A., Pons J., Merkoçi A. (2012). Controlled formation of nanostructured CaCO_3_–PEI microparticles with high biofunctionalizing capacity. J. Mater. Chem..

[23] Volodkin D. (2014). CaCO_3_ templated micro-beads and -capsules for bioapplications. Adv. Colloid Interface Sci..

[24] Parakhonskiy B.V., Foss C., Carletti E., Fedel M., Haase A., Motta A., Migliaresi C., Antolini R. (2013). Tailored intracellular delivery via a crystal phase transition in 400 nm vaterite particles. Biomater. Sci..

[25] Manabe K., Oniszczuk J., Michely L., Belbekhouche S. (2020). pH- and redox-responsive hybrid porous CaCO_3_ microparticles based on cyclodextrin for loading three probes all at once. Colloids Surf. A Physicochem. Eng. Asp..

[26] Belbekhouche S., Bousserrhine N., Alphonse V., Carbonnier B. (2019). From beta-cyclodextrin polyelectrolyte to layer-by-layer self-assembly microcapsules: From inhibition of bacterial growth to bactericidal effect. Food Hydrocoll..

[27] Haložan D., Riebentanz U., Brumen M., Donath E. (2009). Polyelectrolyte microcapsules and coated CaCO_3_ particles as fluorescence activated sensors in flowmetry. Colloids Surf. A Physicochem. Eng. Asp..

[28] Antipov A.A., Shchukin D., Fedutik Y., Petrov A.I., Sukhorukov G.B., Möhwald H. (2003). Carbonate microparticles for hollow polyelectrolyte capsules fabrication. Colloids Surf. A Physicochem. Eng. Asp..

[29] Michely L., Chesneau C., Dika E., Evrard T., Belbekhouche S. (2022). Easy way for fabricating calcium carbonate hybrid microparticles-supported carrier: Focus on the loading of several hydrosoluble cargos all at once. J. Drug Deliv. Sci. Technol..

[30] Hobbs S.K., Monsky W.L., Yuan F., Roberts W.G., Griffith L., Torchilin V.P., Jain R.K. (1998). Regulation of transport pathways in tumor vessels: Role of tumor type and microenvironment. Proc. Natl. Acad. Sci. USA.

[31] Zhao D., Zhuo R.-X., Cheng S.-X. (2012). Alginate modified nanostructured calcium carbonate with enhanced delivery efficiency for gene and drug delivery. Mol. Biosyst..

[32] Hu Q.-D., Tang G.-P., Chu P.K. (2014). Cyclodextrin-Based Host–Guest Supramolecular Nanoparticles for Delivery: From Design to Applications. Accounts Chem. Res..

[33] Uekama K., Hirayama F., Irie T. (1998). Cyclodextrin Drug Carrier Systems. Chem. Rev..

[34] McCormack B., Gregoriadis G. (1994). Entrapment of Cyclodextrin-Drug Complexes into Liposomes: Potential Advantages in Drug Delivery. J. Drug Target..

[35] Belbekhouche S., Oniszczuk J., Pawlak A., El Joukhar I., Goffin A., Varrault G., Carbonnier B. (2019). Cationic poly(cyclodextrin)/alginate nanocapsules: From design to application as efficient delivery vehicle of 4-hydroxy tamoxifen to podocyte in vitro. Colloids Surf. B Biointerfaces.

[36] Kurkov S.V., Loftsson T. (2013). Cyclodextrins. Int. J. Pharm..

[37] Suzuki I., Obata K., Anzai J.-I., Ikeda H., Ueno A. (2000). Crown ether-tethered cyclodextrins: Superiority of the secondary-hydroxy side modification in binding tryptophan. J. Chem. Soc. Perkin Trans..

[38] Zhu X.L., Wang H.B., Chen Q., Yang W.C., Yang G.F. (2007). Preparation and characterization of inclusion complex of iprodione and beta-cyclodextrin to improve fungicidal activity. J. Agric. Food Chem..

[39] Chen G., Jiang M. (2011). Cyclodextrin-based inclusion complexation bridging supramolecular chemistry and macromolecular self-assembly. Chem. Soc. Rev..

[40] Loftsson T., Duchêne D. (2007). Cyclodextrins and their pharmaceutical applications. Int. J. Pharm..

[41] Brewster M.E., Loftsson T. (2007). Cyclodextrins as pharmaceutical solubilizers. Adv. Drug Deliv. Rev..

[42] Wei H., Shen Q., Zhao Y., Wang D.-J., Xu D.-F. (2003). Influence of polyvinylpyrrolidone on the precipitation of calcium carbonate and on the transformation of vaterite to calcite. J. Cryst. Growth.

[43] Wang C., He C., Tong Z., Liu X., Ren B., Zeng F. (2006). Combination of adsorption by porous CaCO_3_ microparticles and encapsulation by polyelectrolyte multilayer films for sustained drug delivery. Int. J. Pharm..

[44] Oniszczuk J., Le Floch F., Mansour O., Alimi M., Le Cœur C., Audard V., Sahali D., Carbonnier B., Pawlak A., Belbekhouche S. (2021). Kidney–Targeted drug delivery systems based on tailor-made nanocapsules. Chem. Eng. J..

[45] Bahrom H., Goncharenko A.A., Fatkhutdinova L.I., Peltek O.O., Muslimov A.R., Koval O., Eliseev I.E., Manchev A., Gorin D., Shishkin I.I. (2019). Controllable Synthesis of Calcium Carbonate with Different Geometry: Comprehensive Analysis of Particle Formation, Cellular Uptake, and Biocompatibility. ACS Sustain. Chem. Eng..

[46] Zhang Y., Ma P., Wang Y., Du J., Zhou Q., Zhu Z., Yang X., Yuan J. (2012). Biocompatibility of Porous Spherical Calcium Carbonate Microparticles on Hela Cells. World J. Nano Sci. Eng..

[47] Dhas N.A., Suslick K.S. (2005). Sonochemical Preparation of Hollow Nanospheres and Hollow Nanocrystals. J. Am. Chem. Soc..

[48] Qiu N., Yin H., Ji B., Klauke N., Glidle A., Zhang Y., Song H., Cai L., Ma L., Wang G. (2012). Calcium carbonate microspheres as carriers for the anticancer drug camptothecin. Mater. Sci. Eng. C.

