# Supplementary material for: Cyclodextrin-Calcium Carbonate Micro- to Nano-Particles: Targeting Vaterite Form and Hydrophobic Drug Loading/Release
Source: Pharmaceutics. 2023 Feb 15;15(2):653. doi: 10.3390/pharmaceutics15020653 (PMC9963295; doi:10.3390/pharmaceutics15020653)
Supplement: Supplementary file 1 [file pharmaceutics-15-00653-s001.zip › pharmaceutics-2207228-supplementary.pdf]

pharmaceutics

Article

Cyclodextrin-Calcium Carbonate Micro- to Nano-particles : Targeting Vaterite Form and Hydrophobic Drug Loading/Release

Cléa Chesneau[1], Alpha Oumar Sow[1], Fadila Hamachi[1], Laurent Michely[1], Séna Hamadi[1], Rémy Pires[1], André Pawlak[2,3] and Sabrina Belbekhouche[1] *

[1] Université Paris Est Creteil, CNRS, Institut Chimie et Matériaux Paris Est, UMR 7182, 2 Rue Henri Dunant, 94320 Thiais, France
[2] Université Paris Est, Faculté de Médecine, UMRS 955, Créteil, F-94010 France.
[3] Université Paris Est Creteil, CNRS, Institut Chimie et Matériaux Paris Est, UMR 7182, 2 Rue Henri Dunant, 94320 Thiais, France
* Correspondence: belbekhouche@icmpe.cnrs.fr, phone: + 331 4978 1149, fax: + 331 4978 1208 .

Supporting information

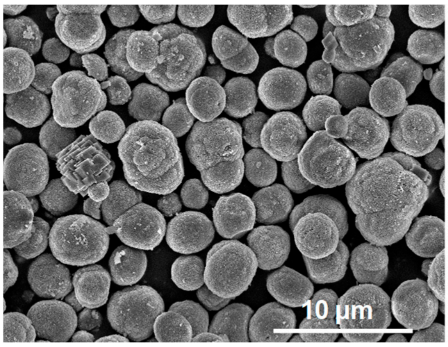

Figure S1. Scanning Electron Microscopy image of CD-calcium carbonate microparticles prepared.
